# Case Report: Invasive Cryptococcosis in French Guiana: Immune and Genetic Investigation in Six Non-HIV Patients

**DOI:** 10.3389/fimmu.2022.881352

**Published:** 2022-04-26

**Authors:** Jeanne Goupil de Bouillé, Loïc Epelboin, Fanny Henaff, Mélanie Migaud, Philippe Abboud, Denis Blanchet, Christine Aznar, Felix Djossou, Olivier Lortholary, Narcisse Elenga, Anne Puel, Fanny Lanternier, Magalie Demar

**Affiliations:** ^1^ Avicenne Hospital, Assistance Publique des Hôpitaux de Paris, Bobigny, France; ^2^ Laboratoire Éducation et Pratique de Santé, University of Sorbonne Paris Nord, Bobigny, France; ^3^ Cayenne Hospital, Cayenne, French Guiana; ^4^ University of French Guiana, Cayenne, French Guiana; ^5^ Imagine Institute, Paris Cité University, Paris, France; ^6^ Laboratory of Human Genetics of Infectious Diseases, Necker Branch, Institut national de la santé et de la recherche médicale U1163, Necker Hospital, Assitance Publique des hôpitaux de Paris (APHP), Paris, France; ^7^ St. Giles Laboratory of Human Genetics of Infectious Diseases, Rockefeller University, New York, NY, United States; ^8^ Unité Mixte de Recherche 2000, Pasteur Institute Paris, University of Paris, Paris, France

**Keywords:** cryptococcosis, immunocompetent, STAT1 gene, autoantibodies against GM-CSF, antibodies against IFN-γ, fungal infection

## Abstract

**Objectives:**

We describe the clinical, mycological, immunological, and genetic characteristics of six HIV-negative patients presenting with invasive cryptococcosis.

**Methods:**

Patients with cryptococcosis without any of the classical risk factors, such as HIV infection, followed at Cayenne Hospital, were prospectively included. An immunologic and genetic assessment was performed.

**Results:**

Five male patients and one female patient, 5 adults and one child, were investigated. All presented a neuromeningeal localization. *Cryptococcus neoformans* var. *gattii* and *C. neoformans* var. *grubii* were isolated in two and three patients, respectively, whereas one patient could not be investigated. Overall, we did not observe any global leukocyte defect. Two patients were found with high levels of circulating autoantibodies against Granulocyte macrophage-colony stimulating factor (GM-CSF), and none had detectable levels of autoantibodies against Interferon gamma (IFN-γ) Sequencing of *STAT1* exons and flanking regions performed for four patients was wild type.

**Conclusion:**

To better understand cryptococcosis in patients with cryptococcosis but otherwise healthy, further explorations are needed with repeated immune checkups and strain virulence studies.

## Highlights

- Invasive cryptococcosis in otherwise healthy individuals is rare.
- This study presents the clinical manifestations and the immune and genetic explorations performed in six of such patients.

## Introduction

Cryptococcosis is a life-threatening fungal infection of immunosuppressed patients, well described in HIV-infected patients. More rarely, it occurs in patients without any of the known classical risk factors. The mechanism of the infection of these patients remains unclear, and we could hypothesize that otherwise healthy individuals with cryptococcosis carry a rare inborn error of immunity affecting specifically their immune response to *Cryptococcus* spp. The particular virulence of certain strains of *Cryptococcus* could also be involved.

The current classification based on its capsule immunologic and molecular analysis reports three varieties, five serotypes, and eight molecular types (1). *Cryptococcus neoformans* var. *grubii* has a worldwide distribution and is mainly responsible for cryptococcosis in patients with acquired immunosuppression (e.g., AIDS), whereas *Cryptococcus gattii*, mainly found in tropical and subtropical areas, usually strikes otherwise healthy individuals (2–4). In addition, anti-GM-CSF antibodies (5, 6) and primary immune deficiencies have been previously associated with crypotococcosis [STAT1 Gain of Function (GOF) (7, 8), STAT3 deficiency (9, 10), CD40 ligand deficiency (11)].

Few cases of cryptococcosis have been reported in French Guiana, a French overseas territory located on the northeastern coast of South America, supposedly in patients who were otherwise healthy. In the current study, we assessed the clinical, epidemiological, mycological, immunological, and genetic characteristics of patients from French Guiana with invasive cryptococcosis without known underlying causes. A better understanding of these features should help for an earlier and better diagnosis, preventing complicated forms of cryptococcal infections, and should bring new insights into the pathogenesis of the disease.

## Materials and Methods

### Study Site

The study was performed at the Cayenne Hospital in French Guiana, a French overseas territory of 250,000 inhabitants, located between Brazil and Suriname in the Amazonian region.

### Study Design

A prospective analysis was carried out on all consecutive non-HIV patients who were admitted to the Cayenne Hospital from 2011 to 2018 and diagnosed with cryptococcosis.

### Case Definition

In accordance with the European Organisation for Research and Treatement of Cancer (ORTC) criteria for invasive fungal infections (12), invasive cryptococcosis was defined by at least one of the following criteria:

i) Histopathologic, cytopathologic, or direct microscopic examination of *Cryptococcus* obtained by needle aspiration or biopsy from a normally sterile site showing yeast cells.
ii) Recovery of a yeast by culture of a sample obtained by a sterile procedure from a normally sterile site showing a clinical or radiological abnormality consistent with an infectious disease process.
iii) Blood culture that yields yeast and cryptococcal antigen in cerebrospinal fluid (CSF).
iv) Amplification of cryptococcal DNA by PCR combined with DNA sequencing.

### Immune Investigation

After inclusion, the patients were evaluated for their immune profile, including the following: i) lymphocyte immunophenotyping, immunoglobulin (IgG, IgA, IgM) levels, complement (CH50, C3, C4) levels, autoimmunity investigation by evaluating the presence of antinuclear antibodies (ANAs), anti-cardiolipin antibodies (ACAs), anti-β2-glycoprotein I antibodies, lupus anticoagulant; ii) the Interleukin (IL)-12/IFN-γ axis exploration, the presence of autoantibodies (auto-Abs) against GM-CSF and IFN-γ; iii) *STAT1* exons and flanking intronic regions were sequenced in four patients.

### Data Collection

Data were collected from patient medical records: i) general demographic data; ii) laboratory data including biochemistry, hematology, immunology, and microbiology; iii) radiology variables and additional investigations depending on the findings; iv) the clinical and therapeutic management; v) the outcome of the patients.

All patients and/or relatives gave informed written consents.

The study received the agreement of the Committee of Protection of the Persons of the University Paris II on September 6, 2010 and of the AFFSAPS under the number B100712-40.

## Results

### Description of Cases

During the study period, six patients were included. Clinical, epidemiological, and fungal characteristics are reported in **Table 1**. Most were male patients (5/6), with a median (minimum–maximum) age of 23.5 years (4–55 years) at the time of inclusion in the study. They were from various origins; one Creole Haitian male patient, one Creole French Guianese male patient, one Hmong male patient (refugee people from Vietnam war), two Brazilian citizens, and an Amerindian male patient. None of the patients had significant past medical history (cf **Table 1**).

**Table 1:** Clinical, epidemiological, fungal characteristics of the 6 *Cryptococcosis* cases.

	Case 1	Case 2	Case 3	Case 4	Case 5	Case 6	MedianIQR
**Age (years)**	17	15	37	30	55	4	23.5 (15.5–35.2)
**Sex**	Male	Male	Male	Female	Male	Male	
**Biotope of the usual residency**	Urban	Semi-natural forest	Urban	Urban	Rural	Primary forest	
**Cultural group**	Haitian	Hmong	French Guianese Creole	Brazilian	Brazilian	Amerindian	
**Medical history**	None	None	Meningitis Steatosis Hypothyroiditis Polyglobulia	Thyroid nodules	None	Asthma	
**Time to diagnosis since the onset of the symptoms (days)**	89	16	57	11	3	30	23 (12.2–50.2)
**Location of infection**	Meningo encephalitis Pulmonary nodule Skin	Meningoencephalitis Hematologic	Meningitis	Meningoencephalitis Cerebral nodules Pulmonary infection	Meningoencephalitis	Meningoencephalitis Pulmonary Nodules	
**Symptoms**	Headache Neck pain Fever	Headache Quadriplegia Blindness Bilateral Hypoacusis Intracranious hypertension	Headaches Vomiting Intracranial hypertension Homonymous hemianopsia	Pulmonary infection Meningitis Intracranious hypertension Blindness Diplopia Scotomas	Fever Headaches Vomiting Intracranious hypertension Confusion Motor deficit Upper right limb Left ptosis	Loss of weigh Cough, Headache, Vomiting, Intracranious hypertension hydrocephalus	
**Sequelae**	None	Blindness Hearing loss Psychomotor retardation	Persistent headaches	Blindness Loss of the sense of smell	Ideomotor slowdown	None	
**Lumbar punction pressure**	Not done	85 mmHg	Not done	25 mmHg	110 mmHg	49 mmHg	67 mmHg (43–91))
**Brain MRI Abnormalities**	Nodular lesions (temporal, frontal, parieto-occipital), Peripheral ring signal Enhancement and Perilesional edema	Periventricular bilateral FLAIR hypersignal	Hyperintensities in the brain’s white matter (supratentorial, cerebellar), ventricular dilatation	Multiple diffuse nodular brain lesions, Perilesional edema	Pachymeningitis Diffuse high-intensity signal	Periventricular hyperintensity, tetra ventricular dilatation	
**Chest CT abnormalities**	Pulmonary nodules of right basal pyramid excavated	None	None	Nodules	None	Pulmonary intraparenchymal cystic formations Parenchymal condensation Excavated nodules	
**CSF Leukocytes (/mm3) and lymphocyte count (%)**	74 92%	180 99%	130 100%	135 70%	236 60%	10 Not realized	132.5 (88–168.7) 92 (70–99)
**CSF sugar (mmol/l) Blood sugar (mmol/l) CSF proteins (g/l)**	2,2 5,8 1,5	2.1 4.3 0.6	0.2 3.9 1.5	3,3 3.3 6.5	1.9 6.5 6.1	1.7 4.5 0.5	2.0 (1.7–2.2) 4.4 (4–5.5) 1.5 (0.8–4.9)
**CSF Antigen titer**	1:10	1:100	1:120	1:10	1:100	Not done	
**CSF culture and identification**	*C. gattii*	*C. neoformans* var. *grubii*	*C. neoformans* var. *grubii*	*Cryptococcus* sp.	*C. neoformans* var. *grubii*	*C. gattii*	3 *C. neoformans* var. grubii *2 C. gattii* 1 *Cryptococcus* Sp.
**Blood antigen titer at diagnosis**	1:20	1:100	1:100	1:10	1:1000	1:640	
**Induction treatment**	Amphotericin B + Flucytosine 31 days	Amphothericin B + Flucytosine 31 days	Amphotericin B + Flucytosine 15 days	Amphotericin B + Flucytosine Duration not known	Amphotericin B + Flucytosine 15 days	Amphotericin B + Flucytosine 15 days	
**Neurosurgery management**	No	Yes	No	No	No	Yes	
**Corticotherapy**	Yes	Yes	No	No	No	Yes	
**Consolidation treatment**	Fluconazole 800 mg/day	Fluconazole 800 mg/day + Flucytosine	Fluconazole 800 mg/day	Fluconazole 800 mg/day	Fluconazole 400 mg/day	Fluconazole 12 mg/kg/day	

MRI, magnetic resonance imaging; CT, computed tomography; CSF, cerebrospinal fluid; sp., species; IQR, Interquartile range; CRP, C reactive protein; NK, Natural Killer; BCG, Bacillus Calmette Guerin; IDSA, Infectious Diseases Society America..

**Figure 1:**
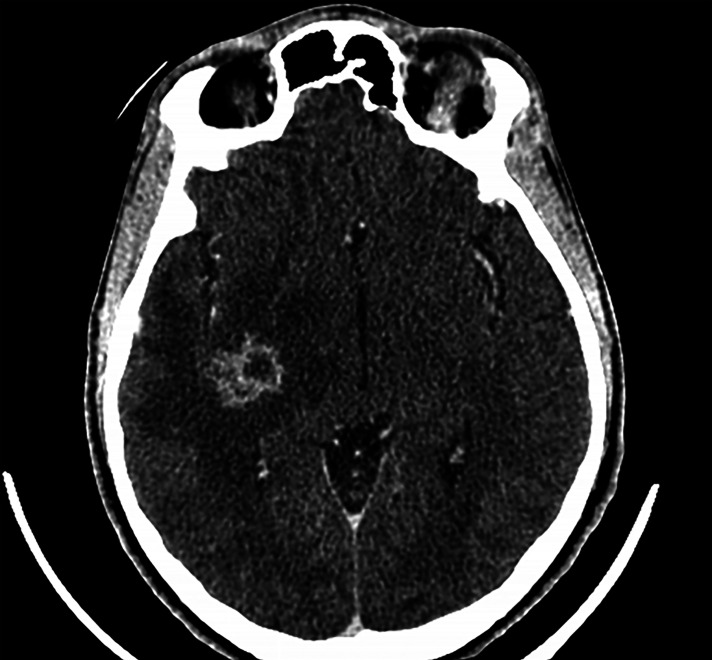
Brain Computed Tomography-scan with nodular, right insular lesion with cocoon enhancement and peri-lesional edema.

**Figure 2:**
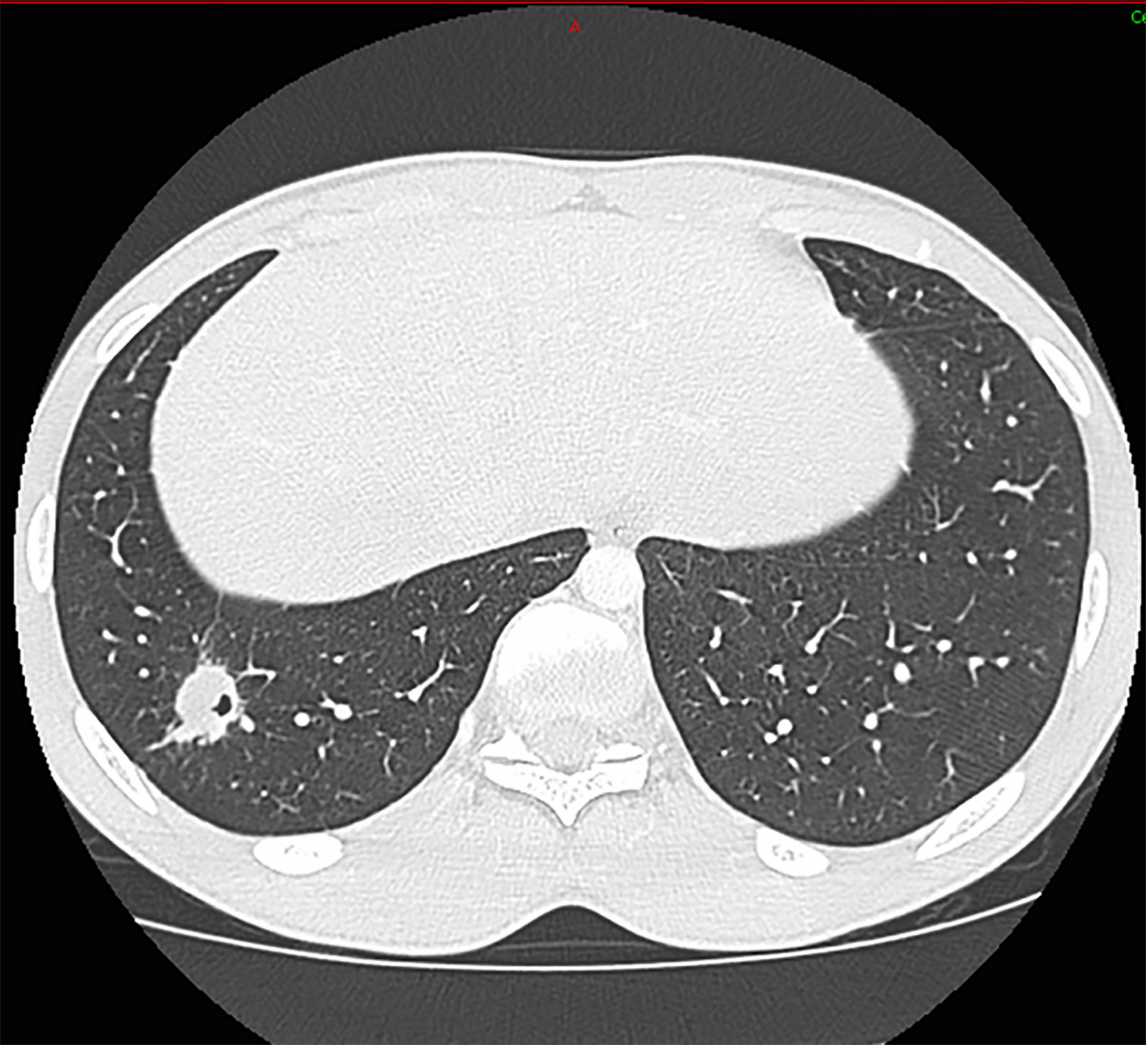
Pulmonary Computed Tomography–scan with pulmonary nodule of the right basad pyramid excavated.

All patients had neurological involvement, mostly meningoencephalitis. One patient was reported with brain cryptococcoma. Another patient suffered from an isolated meningitis without encephalitis. The neurological presentation manifested either as an uncomplicated symptom type (headache or neck stiffness) or in a more severe form as motor deficit, confusion. One patient had vigilance disorders requiring intensive care monitoring. Clinical signs of intracranial hypertension were present for all patients except one. Ophthalmologic disorders such as blindness or diplopia were found in four patients. The median (IQR) CSF opening pressure was 67 mmHg (43–91 mmHg). Median cellularity of CSF was 132.5 (88.0–168.7) leukocytes/mm^3^ with lymphocytic predominance. Five patients presented abnormalities on their brain imaging (MRI and/or CT scan) when included (**Figure 1**). Three cases presented, in addition, pulmonary involvement with nodules (**Figure 2**). Pulmonary symptomatology was often absent, and the lesions were revealed during complimentary tests. Only one patient had a cough.

All patients received induction therapy with amphotericin and flucytosine, 3 received corticosteroid therapy, and 2 had neurosurgical management. Consolidation therapy always included fluconazole. One of the patients also had flucytosine.

Neurological sequelae were common (four out of six patients) ranging from chronic headaches to blindness and deafness.

The serotyping led to the identification of three *C. neoformans* var. *grubii* and two *C. gattii.* The identification of *Cryptococcus* serotype could not be specified for one patient. The time to diagnosis was longer for *C. gattii* vs. *C. grubii* serotypes. One microbiological sampling was performed to isolate *Cryptococcus* in the lung (case 1) and was positive. Pulmonary lesions were found only *for C. gattii* species.

### Immune Exploration

Immune explorations of the cases are reported in **Table 2**. None of the patients presented any remarkable increase of inflammatory markers in their serum with a median (IQR) CRP of 5.8 mg/L (2.7–9.4 mg/L). All patients were HIV and HTLV1 seronegative, and none received any immunosuppressive therapy. Immunoglobulin levels were normal in all patients. All patients had normal global lymphocyte immunophenotyping, except case 2, who showed a transient low NK cell count at the time of infection, which was fully restored a few months after the acute episode.

**Table 2:** Immune exploration of the *Cryptococcosis* cases.

	Case 1	Case 2	Case 3	Case 4	Case 5	Case 6
**HTLV1 serology**	Negative	Negative	Negative	Negative	Negative	Negative
**Lymphocyte immunophenotyping** **(CD4/CD8/B/NK)**	Normal	NK lymphopenia Re-controlled Normal	Normal	Normal	Normal	Normal
**Immunoglobulin (g/g/L)**	Normal	Normal	Normal	Normal	Normal	Normal
**Study of IL12/Interferon gamma production** **(in comparison with a healthy individual)**	Normal	Normal	Normal	Not done	Not done	Normal
**Anti-GM-CSF antibodies**	Positive with positive neutralizing activity	Negative	Negative	Positive with positive neutralizing activity	Not done	Negative
**Anti-IFN γ antibodies**	Negative	Negative	Negative	Not done	Not done	Negative
**STAT1 gene**	Not done	Wild type	Wild type	Wild type	Not done	Wild type

**Author Bio**: Dr. Jeanne Goupil is an infectious disease specialist. She is currently practicing in the suburbs of Paris. Her research interests include tropical diseases, HIV, and public health.

Anti-nuclear factors were found in only one patient, with titers in the upper limit of normal values (1/80). Circulating anticoagulants were found to be transiently positive in one patient but became negative on a second test. Two patients had anti-cardiolipin antibodies. Complement (C4, CH50, C3) levels were consistently normal. Whole blood activation from four patients with BCG without or with IL-12 or IFN-γ showed normal production of IFN-γ or IL-12, respectively, suggesting a normal IL-12/IFN-γ axis. Two out of five patients tested showed high titers of neutralizing auto-Abs against GM-CSF. All *STAT1* exons were sequenced for four patients and were found to be wild type.

## Discussion

Cryptococcosis is a well-known fungal infection in French Guiana. A retrospective study, conducted between 1998 and 2008, identified 43 patients with cryptococcosis admitted to hospitals in French Guiana (13). Fourteen cases (32.6%) were not infected with HIV, and of these 14 patients, only 2 (4.7%) had another detected cause of immunosuppression (corticotherapy alone or associated with diabetes mellitus). Whereas the sex ratio (M/F) was equal to 1 in the HIV-negative group, M/F ratio was 2.63 among the 29 HIV-positive patients. Patients of the HIV-negative group were older (51.6 ± 23.9) than those of the HIV-positive group (41.8 ± 12.5). The average incidence of cryptococcosis was estimated at 22.6 cases/million inhabitants/year during the period 1998–2008, about 10 times higher than in metropolitan France (14).

The clinical presentations of our patients were similar to those of cases reported in the literature. Unlike patients with immunodeficiency, cerebral and pulmonary forms are predominant in patients with cryptococcosis but otherwise healthy (13, 15–18). They frequently present as indolent forms of meningitis, and visual symptoms are the most frequent manifestations (13). In rare cases, the neurologic symptomatology can be very noisy and be associated with high and recurrent neurological morbidities, including shunt requirements, serial lumbar punctures, and pressure-related complications (19). The mean time at diagnosis is significantly longer. CSF white blood cell counts are usually higher, and meningeal enhancement on CT scan of the brain is more frequently observed (16, 19). Cryptococcaemia and other extraneural, extrapulmonary (digestive, ganglionic, oropharyngeal, dermatologic, hematologic, or bone) manifestations are much more associated with immunodeficiency and poorer prognosis (18, 20–23).

In this study, we identified three patients with *C. neoformans* var. *grubii* and two patients with *C. gattii*. A French Guianese study showed an epidemiology for *Cryptococcus* composed mainly with A (77.3%) and B (22.7%); no types C and D were revealed. This differs from a study performed in France, especially for types B and D showing respectively 1.8% and 22.9%. *C. neoformans* var. *gattii* usually invades more frequently the brain and pulmonary parenchyma and causes multiple granuloma (24–26). Cryptococcomas can appear in any region of the lung and can have different sizes. Visual alterations are also more frequent in *C. gattii* (27).

We found anti-GM-CSF auto-Abs in two patients, as previously reported in otherwise healthy patients with cryptococcal meningitis caused by *C. gattii* (5, 6). One of the patients was infected with *C. gattii*, whereas we could not test the strain in the other one. These auto-Abs were neutralizing, as shown *in vitro*, with an abolished STAT5 phosphorylation upon GM-CSF stimulation of control peripheral blood mononuclear cells (PBMCs) in the presence of 10% of patients’ plasma, but not in the presence of 10% of healthy individuals’ plasma, probably inhibiting macrophage function *in vivo*. We also tested for the presence of anti-IFN-γ auto-Abs, as some patients with cryptococcosis were also reported with such auto-Abs (28–30); none of the 6 patients tested displayed anti-IFN-γ auto-Abs.

In addition, few inborn errors of immunity have been associated with increased susceptibility to cryptococcosis [CD4 lymphopenia (31), X-linked CD40L deficiency (11), STAT3 mutated hyper-IgE syndrome (9, 10)]. Among them, some patients carrying heterozygous STAT1 GOF mutations (7, 8) were found with cryptococcosis (32). However, none of the four patients tested in our cohort carried any rare variants of *STAT1*. In our studies, no ethnic group was overrepresented such as aborigines in Australia (33) suggesting that genetic factors may be important.

Some limitations should be considered in this study. Due to logistical difficulties in delivering immunoassay and lost to follow-up, the study has missing data. Genetic studies have only screened rare variants of *STAT1*. A particular virulence of the strain could be evoked (24, 34, 35). Unfortunately, this parameter could not be studied in this study.

Our study highlights the difficulty of determining the causal agent of cryptococcosis in patients. It thus opens up different avenues for consideration. The immune status of the host and a particular virulence of the *Cryptococcus* strain are the two main hypotheses.

**Figure 3:**
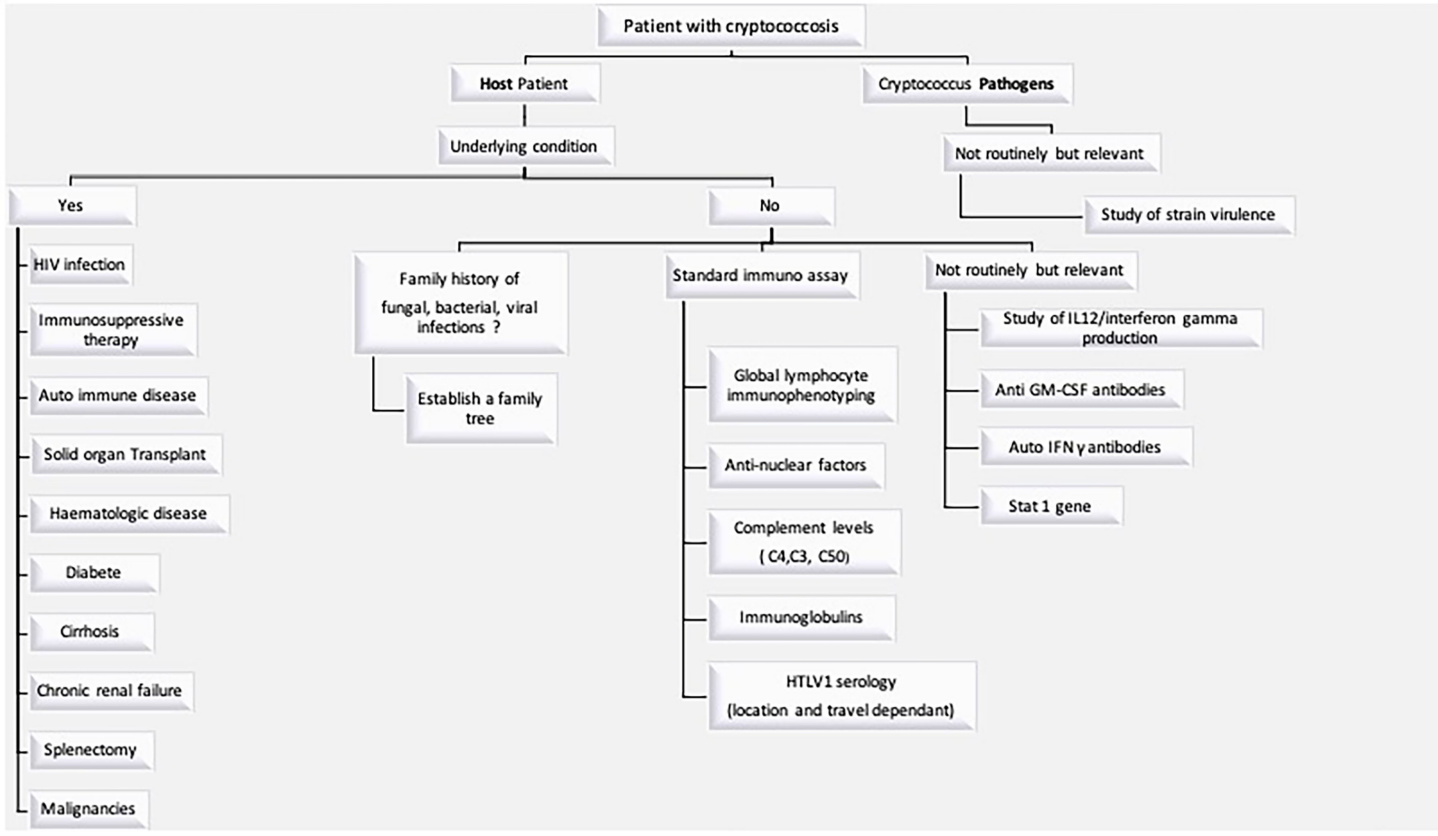
Investigation to be performed in a patient with cryptococcosis.

The immune status of the host is a key issue, since, as notified in IDSA Guidelines (25), treatment depends on it. A comprehensive immune and genetic exploration, in our opinion, is the first step in answering the various questions. To our knowledge, the present study is the first that proposes a standardized and detailed immunological assessment for so-called “immunocompetent” patients suffering from cryptococcal disease (**Figure 3**). It seems unclear whether these patients have phenotypic or genetic deficits. Genetic analyses are not easy to carry out routinely but must be integrated into research programs. It could also be assumed that cryptococcosis is the cause of immunosuppression. In case 2, a dosage of lymphocyte NK was abnormal during the infection. A control was performed a few months later with normal proportion of lymphocyte NK. This suggests that *Cryptococcus* infection can suppress the immune system, and its elimination contributes to the reestablishment of an immune equilibrium. French Guiana is known for its specificities in terms of tropical infections, and we could evoke the virulence of a specific strain in our patients. Further *in vivo* investigation is essential to understand the basic mechanism of virulence of *C. gattii* and *C. grubii* especially in tropical areas where epidemiology is different from the other areas (36–40).

## Conclusion

This study describes the clinical, biological, immunological, and genetic characteristics of six non-HIV patients in French Guiana suffering from cryptococcosis. Clinical presentations can be devious, and they highlight the particularities of this infection according to the *gattii* or *grubii* serotype. Cryptococcosis is a potentially emerging disease. Two out of the six patients tested had high titers of neutralizing auto-Abs against GM-CSF, and this consequent percentage deserves further studies on these antibodies. None of the four patients tested carry rare variants of *STAT1*, the only candidate gene tested yet. Studying patients with cryptococcosis but otherwise healthy should help to progressively decipher the crucial physiopathological mechanisms underlying this disease.

## Data Availability Statement

The original contributions presented in the study are included in the article/supplementary material. Further inquiries can be directed to the corresponding author.

## Ethics Statement

The studies involving human participants were reviewed and approved by the Committee of Protection of the Persons of the University Paris II on 2010-06-09 and of the AFFSAPS under the number B100712-40. Written informed consent to participate in this study was provided by the participants’ legal guardian/next of kin.

## Author Contributions

Conceptualization: JGdB and MD. Formal analysis: JGdB. Investigation: JGdB, LE, FH, MM, FL, PA, DB, AP, CA, and OL. Methodology: JGdB and MD. Supervision: MD. Writing—original draft: JGdB. Writing—review and editing: JGdB, LE, FH, MM, PA, DB, CA, FD, NE, AP, FL, and MD. All authors have read and approved the final article.

## Funding

The work was funded by the French National Research Agency (ANR) under the “Investments for the future” program (ANR-10-IAHU-01), the ANR-FNS LTh-MSMD-CMCD (ANR-18-CE93-0008-01), the Integrative Biology of Emerging Infectious Diseases Laboratory of Excellence (ANR-10-LABX-62-IBEID), and the National Institute of Allergy and Infectious Diseases of the NIH (grant no. R01AI127564).

## Conflict of Interest

The authors declare that the research was conducted in the absence of any commercial or financial relationships that could be construed as a potential conflict of interest.

## Publisher’s Note

All claims expressed in this article are solely those of the authors and do not necessarily represent those of their affiliated organizations, or those of the publisher, the editors and the reviewers. Any product that may be evaluated in this article, or claim that may be made by its manufacturer, is not guaranteed or endorsed by the publisher.
